# Testing the Integral Model of treatment motivation in outpatients with severe mental illness

**DOI:** 10.1007/s11031-018-9708-0

**Published:** 2018-06-15

**Authors:** E. C. Jochems, H. J. Duivenvoorden, A. van Dam, C. L. Mulder, C. M. van der Feltz-Cornelis

**Affiliations:** 1 Department of Psychiatry, Erasmus MC University Medical Center, Epidemiological and Social Psychiatric Research Institute, Rotterdam, The Netherlands; 2 GGz Breburg, Lage Witsiebaan 4, 5042 DA Tilburg, The Netherlands; 3 Erasmus MC University Medical Center, Rotterdam, The Netherlands; 4 Tranzo Department, Faculty of Social Sciences, Tilburg University, Tilburg, The Netherlands; 5 GGZ West North Brabant, Bergen op Zoom, The Netherlands; 6 Department of Parnassia Psychiatric Institute, BavoEuropoort, Rotterdam, The Netherlands; 7 Mental Health and Addiction Research Group, University of York, York, UK

**Keywords:** Motivation, Theory, Structural equation model, Psychotic disorders, Personality disorders

## Abstract

The current study tested the Integral Model of treatment motivation (IM) in a sample of 294 outpatients with severe mental illness, using structural equation modelling. The obtained structural model was not consistent with original theory, nor was the model invariant across time and patient groups (psychotic disorders and personality disorders). The patient’s perceived suitability of treatment, perceived costs of treatment and outcome expectancy were most strongly associated with motivation and treatment engagement. The model explained between 22 and 86% of variance in clinical outcomes, depending on the timing of the assessment. Currently, the IM does not constitute a robust framework for patterns through which patients become motivated to engage in treatment, but does explain substantial amounts of variance in clinical outcomes. The future potential of IM as a basis for interventions in the mental health care is discussed, including suggestions for subsequent research and potential alterations of the IM to improve its utility for application in clinical practice.

**Electronic supplementary material:**

The online version of this article (10.1007/s11031-018-9708-0) contains supplementary material, which is available to authorized users.

## Introduction

### Background and rationale

The Integral Model of treatment motivation (IM) is a health behavior theory that was specifically developed for application in mental health treatment to understand patients’ motivation for engaging in treatment (Drieschner et al. 2004). The IM holds that six cognitive and emotional factors, called internal determinants (Drieschner et al. 2004), predict the patient’s motivation for engaging in treatment (MET). The patient’s motivation is seen as the mediator between the internal determinants and actual treatment engagement. The Treatment Motivation Scales for forensic outpatient treatment (TMS-f) was developed by the founders of IM to assess the constructs in the theory (Drieschner and Boomsma 2008a). A series of studies using the TMS-f in a forensic psychiatric setting showed support for its hypothesized factorial structure and showed adequate reliability and validity(Drieschner and Boomsma 2008a, b). The studies also found support for the general tenets of the IM, such that three out of six internal determinants were indeed statistically significantly related to the patient’s motivation for engaging in treatment, which in turn was predictive of treatment engagement (Drieschner and Boomsma 2008a, b; Drieschner and Verschuur 2010). However, the relationships between the core constructs of the IM are in need for further empirical testing, including the plausibility and utility of the model outside a forensic psychiatric population. Therefore, the current study aimed to test the IM in a sample of Dutch adult outpatients with severe mental illness using a slightly adapted version of the TMS-f. The following describes the general tenets of IM, previous research findings and our study objectives.

### The Integral Model of treatment motivation

The IM is theoretically affiliated with Ajzen and Fishbein’s theory of planned behaviour (Ajzen 1991), with a strong focus on attitudes toward the behaviour, subjective norms, and perceived behavioural control. The theory of planned behaviour however, does not account for other factors that can influence motivation, such as distress, past experience or environmental factors, which are relevant in the context of motivation for engaging in treatment (-related behaviours) (Drieschner et al. 2004). The IM does take into account these factors more explicitly and may therefore be more useful in the context of mental health care. The IM holds that the patients’ MET depends on the six internal determinants (IDs), which in turn are determined by external factors such as treatment characteristics, external circumstances, life events, demographic features and the type of problem. These external factors are thought to have their effect on motivation only through the IDs (Drieschner et al. 2004). The IDs comprise problem recognition, distress, perceived costs of the treatment, perceived suitability of the treatment, outcome expectancy and perceived legal pressure. *Problem recognition* refers to the recognition that one has a problem, the willingness to admit to the presence of a problem and the recognition that one must change to prevent recidivism. *Distress* is the level of suffering that might result from symptoms, social problems or having fear of deterioration in any area of life. *Perceived costs of the treatment* are the fee and the time the patient feels is spent on treatment, and the psychological costs resulting from exposure to unpleasant emotions and changes in lifestyle. *Perceived suitability of the treatment* encompasses three facets: the patient’s perception of appropriateness and effectiveness of the therapy, the patients’ agreement with the goals of treatment and the patients’ perception of the therapeutic relationship. *Outcome expectancy* refers to the patient’s expectancy of being able to finish the treatment, have success and believe in the ability to change. Finally, *perceived legal pressure* is the patient’s perception of the external pressure through the legal system. As the current study will explore whether the IM is also applicable outside a forensic psychiatric setting, the current study decided to adapt the construct of perceived legal pressure into a more broad *perceived external pressure* by others. This adjustment can be justified by considering that only a subgroup of outpatients with SMI will be referred to or pressured into psychiatric treatment via the legal system, while (most) others will likely experience other pressures that drive their motivation for engaging with treatment (i.e. family, friends, partner, assertive outreaching clinicians). For clarity, we will refer to the revised scale as the TMS-p instead of the TMS-f, to indicate that the revised scale may be applied in a general psychiatric (hence the “p”) population.

Further, MET is thought to predict treatment engagement, which in turn is a predictor of treatment outcome. However, the relationship between MET and treatment engagement is not presumed perfect, because of the possibility that patients may lack the capacity to do what the treatment requires due to cognitive, neuropsychological and other limitations (Drieschner et al. 2004). Also, treatment outcome may depend on the effectiveness of the treatment approach and the persistence of the patients’ problems (Drieschner et al. 2004; Drieschner and Verschuur 2010) which may result in only a modest relationship between treatment engagement and treatment outcome.

**Fig. 1:**
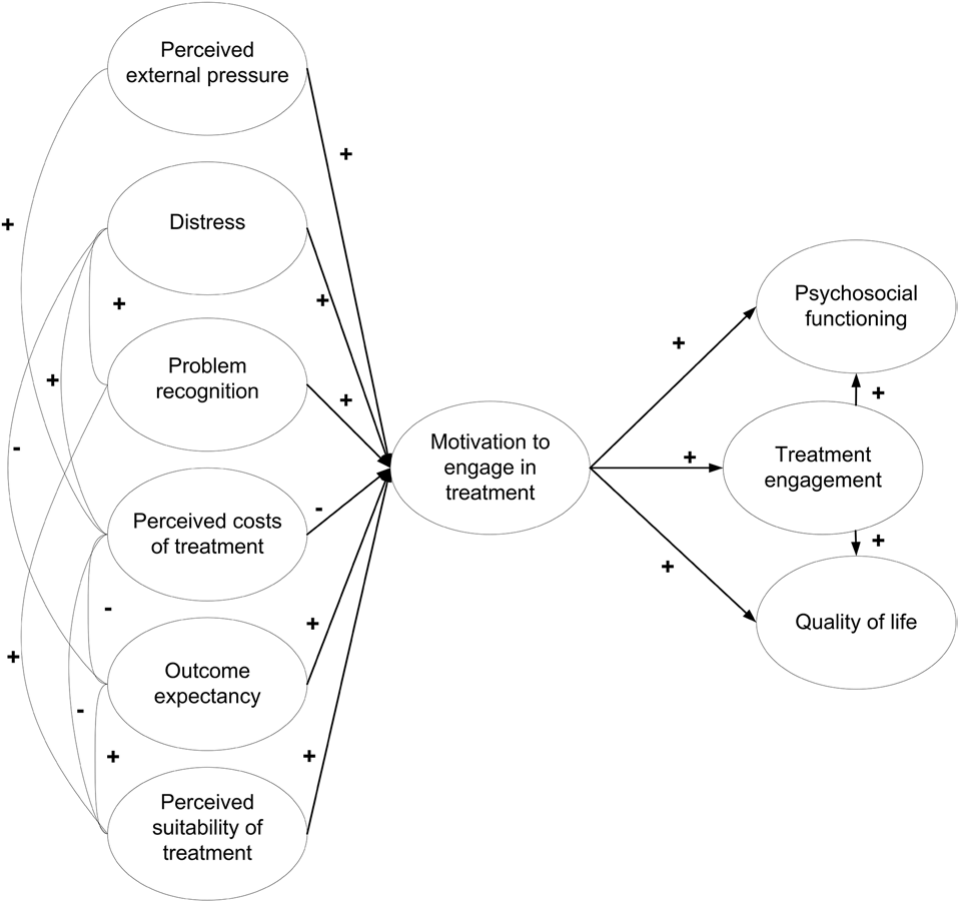
Hypothesized process model for IM. *Note* The figure depicts latent variables, the observed variables and accompanying measurement errors underlying the latent variables were left out to avoid a cluttered presentation

In a previous empirical study on the IM by Drieschner and Boomsma (2008b), the model was mostly supported but not all findings were in line with original hypotheses. For example, treatment engagement was best predicted by MET and by the patient’s perceived suitability of treatment, while distress and perceived legal pressure were found virtually unrelated to MET and treatment engagement (Drieschner and Boomsma 2008b). Also, perceived suitability of treatment was found to predict treatment engagement directly, beyond the mediated effect of MET (Drieschner and Boomsma 2008b). Figure 1 shows the IM as applied to the current study, which is similar to the originally hypothesized model by Drieschner et al. (2004) but includes additional clinical outcomes and perceived external pressure as one of six IDs as opposed to perceived legal pressure. These outcomes were added to evaluate the clinical utility of the model in outpatients with SMI.

The use of scales of measurement requires that they are able to measure the same construct in different populations and at different times, which is called invariance across populations and across time, respectively (Bontempo et al. 2011). That is, although patients may change in their respective levels of motivation and outcome expectancy and perceived external pressure over time, the associations between the constructs in the theoretical model should remain constant across different patient populations and time. To evaluate a motivation theory such as the IM, we argued that a good theory is invariant across populations and across time, such that the theory allows to explain clinical outcomes. Therefore, the current study aimed to test the invariance of IM across time and across patient diagnostic groups, and it was tested whether the model could explain variance in the clinical outcomes psychosocial functioning and quality of life. Specifically, the objectives for the current study were as follows:

### Objectives

1. It was tested whether the IM-model as outlined in Fig. 1 was plausible. We hypothesized that the model in Fig. 1 would show good fit to the data, and if not, we would test which alternative model was most plausible.
2. It was tested whether the most plausible model could be considered invariant across time (i.e. baseline and one year later) and across patient groups (i.e. patients with primarily a personality disorder versus those with primarily a psychotic disorder).
3. The clinical utility of the model was evaluated by investigating to which extent the IM model explained observed variance in the clinical outcomes of psychosocial functioning and quality of life.

## Methods

### Study design

The current longitudinal study constitutes a secondary analysis of a cluster randomized clinical trial (Jochems et al. 2012). The design of this trial and the intention-to-treat analyses were reported elsewhere (Jochems et al. 2012). The current study was approved by the Medical Ethical Committee for Mental Health Care Institutions (Dutch Trial Registry NTR2968) as well as by the scientific committees of the two specialty mental health institutions where the data were collected.

### Setting

Data were collected from 12 outpatient treatment programs, including a forensic psychiatric outpatient clinic, three specialized psychotic outpatient treatment programs and eight function-assertive community treatment teams [FACT-teams (van Veldhuizen 2007)] of two Dutch treatment centres: GGZ Westelijk Noord Brabant and GGz Breburg. FACT-teams provide assertive, outreaching, community-based, and supportive psychiatric services to individuals with SMI (van Veldhuizen 2007), such as those with psychotic disorders and severe personality disorders.

### Participants and procedures

Inclusion criteria for patients were: a primary diagnosis of psychotic or personality disorder, aged 18 to 65 years, undergoing individual outpatient treatment and having a sufficient command of the Dutch language. A clinician was eligible for participation if he or she was the primary health care provider involved with the patient and saw the patient most frequently. Eligible patients on the clinicians’ caseload lists were approached and informed by researchers and asked for their signed consent. Both patients and clinicians were asked to fill in questionnaires at baseline and follow-up assessment (12 months after baseline) and additionally, patients were interviewed regarding their functioning in several life domains by independent research assistants at these assessment moments. To enhance the likelihood of participation, patients were given an incentive of 15 euro for the baseline and follow-up assessment in the trial.

### Measures

#### Core theoretical constructs of IM: internal determinants and motivation for engaging in treatment

Treatment motivation and the six internal determinants were measured by a revised version of the Treatment Motivation Scales for Forensic patients (TMS-f), which we will refer to as the TMS-p. In the TMS-p, the subscale of perceived legal pressure from the TMS-f was adapted to represent a broader perceived external pressure by others. For example, where in the original TMS-f an item is ‘I feel a strong pressure from the legal system’, this was substituted for ‘I feel a strong pressure from others’. The entire modified scale and additional psychometric properties of the adapted subscale can be found in the online supplementary material. Besides the changes to the perceived legal pressure scale, no other changes were made to the original TMS-f. The items were rated on a 5-point Likert scale (0 = totally agree to 5 = totally disagree). The subscale scores were calculated in such a way that a higher score on the subscale represented higher perception of that respective scale, including the subscale perceived costs of the treatment (i.e. higher scores represented higher perceived costs of the treatment). The congeneric estimates of reliability for the seven subscales of the TMS-p for the baseline and follow-up assessment in the current study were as follows: problem recognition = 0.80 and 0.80, distress = 0.90 and 0.91, external pressure = 0.61 and 0.68, costs of treatment = 0.77 and 0.79, suitability of treatment = 0.86 and 0.89, outcome expectancy = 0.86 and 0.85, and motivation for engaging in treatment = 0.82 and 0.86, respectively. The TMS-f was found to be a reliable and valid operationalisation of the constructs in the Integral Model in previous studies (Drieschner and Boomsma 2008a, b). In a previous study in outpatients with severe mental illness, we found statistically significant low to moderate correlations between the motivation subscale of the TMS-p and motivation scales derived from other motivation theories (Jochems et al. 2014).

#### Clinical outcomes: treatment engagement, psychosocial functioning and quality of life

Treatment engagement was measured with the service engagement scale (SES) that was filled out by clinicians. The SES was developed to measure engagement with community mental health services (Tait et al. 2002). It comprises 14 items that assess availability, collaboration, help seeking and treatment engagement behaviours, including medication adherence. The items are rated on a 4-point scale ranging from 0 (not at all) to 3 (most of the time). The SES has good internal consistency (Cronbach’s α = 0.87, congeneric estimate of reliability = 0.91 in the current patient sample) and validity is supported by discrimination between criterion groups (Tait et al. 2002) and significant associations with therapeutic alliance and motivation for engaging in treatment (Jochems et al. 2014). The SES total scale score was used as the outcome measure in this study, where higher scores denote higher treatment engagement.

The patient’s psychosocial functioning was measured with the Dutch version of the Health of the Nations Outcome Scales (HoNOS)(Mulder et al. 2004; Wing et al. 1998). The HoNOS is a semi-structured interview with the patient in which health and social problems of the previous 2 weeks are quantified. It contains 12 items that refer to behavioural problems, cognitive and physical impairments, symptoms, and social functioning. HoNOS items are scored on a scale from 0 (no problem) to 4 (severe problem). The total scale score is computed by adding the 12 items. For ease of interpretation, we reversed the total score such that higher scores reflected higher levels of psychosocial functioning. The administration of the HoNOS was performed by independent research assistants (mostly graduate students in psychology and medicine) who had no involvement in the patient’s treatment. Patients were interviewed at the team office or at home, depending on their preference. The psychometric properties of the total scale score were shown to be acceptable and sensitive to change (Wing et al. 1998). Internal consistency was acceptable in the current study (Cronbach’s α = 0.70, congeneric estimate of reliability = 0.77).

The patient’s quality of life was assessed with the Manchester Short Assessment of Quality of Life (MANSA) (Bjorkman and Svensson 2005; Priebe et al. 1999). The MANSA is a self-report questionnaire that asks the patient how satisfied he/she is in the following life domains: living situation, social relationships, physical health, mental health, safety, financial situation, work situation and life as a whole. The 12 items are scored on a Likert scale from 1 (couldn’t be worse) to 7 (couldn’t be better), which are summed to calculate a total score. Higher scores denote a higher perceived quality of life. The scale is shown to be reliable (i.e. Cronbach’s α = 0.82 and congeneric estimate of reliability = 0.92 in the current patient sample) and other psychometric properties are considered satisfactory (Priebe et al. 1999).

#### Socio-demographic factors and clinical diagnosis

The DSM-IV diagnosis as made by the psychiatrist of the team was obtained from the patients’ medical record, as well as socio-demographic data such as gender, age, ethnicity, age of onset and legal status. If these data were missing in the medical record, the patient was asked to provide this information.

### Statistical analyses

The analyses were performed in several steps. First, the bivariate relations of variables were estimated using Spearman correlations. Structural equation modelling (SEM) as implemented in Mplus version 7.3 (Muthén and Muthén, 1998–2012) was used to test the hypothesized relationships between autonomy support, perceived competence, types of motivation, treatment engagement, psychosocial functioning and quality of life as depicted in Fig. 1.

#### Latent variables

Both at baseline and at follow-up we evaluated the plausibility of the IM-model using latent path analysis as outlined in Fig. 1. We first estimated the congeneric reliabilities (Jöreskog 1971; Reuterberg and Gustafsson 1992) by applying confirmatory factor analyses to the observed items of the following four questionnaires individually: TMS-p, SES, HoNOS and MANSA. Then, the latent constructs for each of these four questionnaires were estimated by a factor analysis model in which the factor loading was fixed at 1.0 and the residual variance of that factor (i.e. 1-congeneric estimate of reliability) was multiplied by the variance of the variable at issue. Hence, the analyses consisted of two steps: (1) observed items of each of the four questionnaires were first added to create total scale scores, (2) then, for each of the four questionnaires, the latent constructs were estimated by correcting the observed total scale scores for unreliability. The latent constructs were used in model testing.

#### Testing the invariance of the structural model

As the type of design was complex (patients clustered within teams) and, in addition, the distributions of the variables were considered to be non-normal, the estimation method used was MLR. This maximum likelihood estimates standard errors and χ^2^ test statistic that are robust to non-normality and non-independence of observations. The MLR standard errors were estimated using a sandwich estimator. Additionally, the variable ‘team’ was included as an additional level in the analyses to adjust for potential clustering of the data within the 12 treatment teams.

First, the model as depicted in Fig. 1 was fitted to the data for the full sample using the baseline and follow-up measurements separately. The following measures were used to test for adequacy of the model fit: χ^2^ for model fit (low and non-significant values of the χ^2^ were desired; *P* value > 0.05); χ^2^/df ratio (a value < 2.0 was considered to be acceptable); information criteria including Akaike (AIC), Bayesian (BIC), sample-size adjusted BIC (SS-BIC) (the smaller the better); comparative fit index (CFI), and Tucker-Lewis Index (TLI) [high values are desired (> 0.95), values > 1.0 point to over identification (Bentler 1990; Tucker and Lewis 1973)]; Root Mean Square Error of Approximation (RMSEA): a value < 0.05 indicates a close fit (Browne and Cudeck 1992); and Standardized Root Mean Squares of Residuals (SRMR: a value of < 0.05 indicates a reliable fit) (Hu and Bentler 1999). Explained variances (R2) were used to describe the performances of the determinants for the individual dependent variables.

It was tested whether the baseline model showed a good overall fit. If not, it was evaluated how it could be adapted such that the fit would improve or alternatively, whether the model could be simplified while not threatening the overall model fit. The most plausible model was obtained by evaluating the model fit criteria and standardized residuals. Further, the MLR χ^2^ difference test was used to compare different models which were nested. The χ^2^ difference was based on log-likelihood values and scaling correction factors obtained with the MLR estimator, using the formula Δχ^2^= − 2* (L0 − L1)/cd where L0 is the log likelihood of the constrained (nested) model, L1 is the log likelihood of the unconstrained model and cd is the difference test scaling correction (which is based on scaling correction factors (c0 and c1) and number of parameters (p0 and p1) for the constrained and unconstrained models, respectively).

The invariance of the most plausible path model across time was evaluated by testing the invariance of the regression estimates of the latent variables, by comparing those assessed at baseline with those assessed at follow-up using the MLR χ^2^ difference test. Fitting both latent path models (baseline and follow-up) jointly was used to test whether the regression estimates of both time points could be considered invariant. Specifically, a non-significant MLR χ^2^ difference test between the model with all regression estimates constrained to be equal for the corresponding measurements versus all regression estimates unconstrained was considered statistical evidence for the latent path model being invariant across time. Individual estimates were regarded statistically significant if the two-sided *P* values were < 0.05. The correlations of the latent variables between the corresponding measurements were allowed to be free as the measurements were repeated. The next step was to test the invariance of the model across different patient groups (personality disorders versus psychotic disorders). This was done according to the same procedure used in testing invariance across time, where the MLR χ^2^ difference test was used to test equality constraints between nested models.

#### Explained variance of clinical outcomes

To test to what extent the obtained IM- model has utility for clinical practice, it was evaluated how much variance was explained on the dependent variables in the model, including treatment engagement, psychosocial functioning and quality of life.

## Results

### Participants and descriptive data

A total of 294 patients and 57 clinicians were included between May 2011 and September 2012. Patient characteristics are shown in Table 1. The majority of patients with psychotic disorders were diagnosed with schizophrenia (48%), schizoaffective disorder (16%), or psychotic disorder not otherwise specified (24%). In the group with primarily personality disorders, 40% had a borderline personality disorder, 13% had antisocial personality disorder, and 26% had a personality disorder not otherwise specified. Most clinicians were female (63%), their mean age was 44 years (SD = 10.70) and they had a mean of 16 years of clinical working experience in mental health services (SD = 9.30). Potentially relevant differences between our study sample and the forensic psychiatric samples studied by Drieschner and Boomsma (2008a, b) include that their sample had a higher percentage of males (around 90%), higher percentage of legal mandates for treatment (around 53%), and less patients with psychotic disorders (around 9%).

**Table 1:** Baseline characteristics of participating patients, stratified by primary diagnosis

	Total patient sample N = 294	Psychotic disorders n = 199	Personality disorders n = 95
Age, mean (SD)	44 (10.3)	43 (10.3)	45 (10.0)
Male gender, n (%)	179 (60.9)	132 (66.3)	47 (49.5)
Dutch ethnicity^a^, n (%)	208 (70.7)	140 (70.4)	68 (71.6)
Education level, n (%)			
No education/elementary	108 (36.7)	76 (38.2)	32 (33.7)
Secondary school	124 (42.2)	75 (37.7)	49 (51.6)
Upper high school and over	59 (20.1)	47 (23.6)	12 (12.6)
Comorbid substance use problems^b^, n (% yes)	74 (25.2)	42 (21.1)	32 (33.7)
Legal mandate, n (% yes)	24 (6.9)	13 (6.5)	11 (12.0)
One or more previous admissions, n, (% yes)	227 (77.2)	159 (79.9)	68 (71.6)
Problem recognition			
Mean (SD)	30.2 (7.7)	28.6 (7.7)	33.75 (6.7)
Min to max (range)	10 to 45 (35)	10 to 45 (35)	16 to 45 (29)
Distress			
Mean (SD)	25.7 (9.6)	23.6 (9.1)	33.8 (6.7)
Min to max (range)	9 to 45 (36)	9 to 45 (36)	12 to 45 (33)
External pressure			
Mean (SD)	30.4 (5.9)	30.2 (6.0)	30.0 (9.2)
Min to max (range)	11 to 45 (34)	11 to 45 (34)	18 to 42 (24)
Perceived costs of treatment			
Mean (SD)	19.9 (6.9)	19.8 (7.1)	30.9 (5.8)
Min to max (range)	9 to 43 (34)	9 to 43 (34)	9 to 37 (28)
Suitability of treatment			
Mean (SD)	35.0 (7.2)	35.1 (7.3)	20.3 (6.4)
Min to max (range)	12 to 45 (33)	14 to 45 (31)	12 to 45 (33)
Outcome expectancy			
Mean (SD)	31.9 (8.1)	32.5 (8.2)	34.7 (7.0)
Min to max (range)	12 to 45 (33)	12 to 45 (33)	13 to 45 (32)
Motivation to engage in treatment			
Mean (SD)	47.2 (11.7)	47.4 (11.7)	46.9 (12.0)
Min to max (range)	16 to 80 (64)	18 to 80 (62)	16 to 78 (62)
Treatment engagement			
Median (IQR)	31 (24 to 36)	32 (25 to 37)	28 (24 to 35)
Psychosocial functioning			
Median (IQR)	9 (6 to 13)	8 (5 to 12)	10 (8 to 15)
Quality of life			
Median (IQR)	5 (4 to 5)	5 (4 to 5)	4 (4 to 5)

*SD* standard deviation, *min to max* minimum value to maximum value on the scale, *IQR* interquartile range

^a^The definition of Dutch Ethnicity was based on the definition by the Dutch Bureau of Statistics

^b^Substance abuse problem was defined as having a DSM-IV diagnosis of substance abuse and/or dependence in the medical record

After 12 months, 253 patients (86%) were re-assessed. The group that was lost to follow-up was significantly more often of non-Dutch ethnicity (48% versus 26%, *P* < 0.01) and more often had a legal mandate for treatment (18% vs. 7%, *P* = 0.03) compared to completers.

Table 2 shows Spearman correlations between variables that were included in the IM model. MET was most strongly correlated with the subscales perceived costs of treatment, suitability of treatment and outcome expectancy. The correlation between motivation for treatment with treatment engagement was moderate for both time points (*r* = .28 and *r* = .30, respectively). Further descriptive statistics of the TMS-p scales, including results from confirmatory factor analyses on each subscale and on the model including the six IDs as predictors for motivation, are presented in the supplementary material online. Based on these analyses, it was decided that the TMS-p was suitable for subsequent analyses in the current study.

**Table 2:** Spearman intercorrelations of variables in the model for the total study sample

	Baseline assessment										Follow-up assessment									
	PR	DS	EP	CT	ST	OE	MET	TE	PF	QL	PR	DS	EP	CT	ST	OE	MET	TE	PF	QL
PR																				
DS	**0.54**																			
EP	**0.54**	**0.28**																		
CT	0.02	**0.35**	0.01																	
ST	**0.12**	**− 0.34**	**0.24**	**− 0.59**																
OE	**−** 0.11	**− 0.55**	0.08	**− 0.61**	**0.68**															
MET	0.10	**− 0.18**	0.07	**− 0.50**	**0.38**	**0.51**														
TE	0.03	**− 0.24**	**0.17**	**− 0.26**	**0.30**	**0.30**	**0.30**													
PF	**− 0.30**	**− 0.56**	**− 0.17**	**− 0.27**	**0.20**	**0.37**	**0.18**	**0.35**												
QL	**− 0.19**	**− 0.57**	**−** 0.02	**− 0.32**	**0.33**	**0.45**	**0.26**	**0.37**	**0.57**											
Follow-up assessment																				
PR	**0.58**	**0.38**	**0.43**	0.02	0.09	**−** 0.05	**0.17**	0.09	**− 0.19**	**− 0.13**										
DS	**0.40**	**0.68**	**0.19**	**0.25**	**− 0.29**	**− 0.40**	**− 0.16**	**− 0.19**	**− 0.37**	**− 0.46**	**− 0.56**									
EP	**0.44**	**0.25**	**0.57**	0.00	**0.16**	0.06	**0.14**	**0.18**	**−** 0.08	**−** 0.05	0.63	**0.37**								
CT	0.05	**0.26**	0.00	**0.58**	**− 0.48**	**− 0.49**	**− 0.34**	**− 0.22**	**− 0.25**	**− 0.35**	0.14	**0.40**	0.04							
ST	0.03	**− 0.28**	**0.19**	**− 0.46**	**0.66**	**0.54**	**0.38**	**0.31**	**0.20**	**0.36**	0.07	**− 0.37**	**0.19**	**− 0.67**						
OE	**−** 0.10	**− 0.42**	0.06	**− 0.43**	**0.51**	**0.61**	**0.33**	**0.23**	**0.27**	**0.41**	**−** 0.25	**− 0.60**	**−** 0.04	**− 0.70**	**0.70**					
MET	0.01	**− 0.16**	0.08	**− 0.37**	**0.36**	**0.38**	**0.61**	**0.28**	**0.17**	**0.27**	**−** 0.02	**− 0.28**	0.04	**− 0.49**	**0.49**	**0.54**				
TE	**0.18**	**−** 0.07	**0.23**	**− 0.26**	**0.28**	**0.24**	**0.26**	**0.63**	**0.21**	**0.25**	0.09	**− 0.13**	**0.23**	**− 0.35**	**0.41**	**0.33**	**0.32**			
PF	**− 0.22**	**− 0.48**	**− 0.14**	**− 0.22**	**0.21**	**0.34**	**0.20**	**0.30**	**0.52**	**0.40**	**−** 0.34	**− 0.60**	**− 0.20**	**− 0.27**	**0.27**	**0.37**	**0.22**	**0.23**		
QL	**− 0.24**	**− 0.52**	**−** 0.11	**− 0.30**	**0.34**	**0.39**	**0.21**	**0.21**	**0.33**	**0.58**	**−** 0.33	**− 0.73**	**− 0.21**	**− 0.39**	**0.38**	**0.49**	**0.25**	**0.19**	**0.61**	

Boldface indicates *P* < 0.05 (two-tailed)

*PR* Problem recognition, *DS* distress, *EP* external pressure, *CT* perceived costs of treatment, *ST* perceived suitability of treatment, *OE* outcome expectancy, *MET* motivation to engage in treatment, *TE* treatment engagement, *PF* psychosocial functioning, *QL* quality of life

### Path analysis

#### Establishing a plausible structural model

First, all observed variables were linearly transformed by a factor of 10 to reduce their variances which allowed Mplus to reach convergence. The observed variables were then corrected for unreliability resulting in the latent variables, which were used in the subsequent path analyses. Table 3 shows the model fit information of the models that were subjected to latent path analyses. The IM- model as depicted in Fig. 1 was fitted to the data at baseline (Model 1a) and at follow-up (Model 2a). Model 1a provided a bad fit to the data (χ^2^/df = 8.30, RMSEA = 0.16, CFI = 0.88, TLI = 0.71, SRMR = 0.13) and Model 2a provided a borderline fit (χ^2^/df = 3.94, RMSEA = 0.10, CFI = 0.95, TLI = 0.86, SRMR = 0.09).

**Table 3:** Model information

Model fit information										Model comparisons with model 1d/2d		
Model	χ^2^	df	χ^2^/df	*P* value	RMSEA	90% CI for RMSEA	CFI	TLI	SRMR	Δ Χ^2^	Δ df	Interpretation based on statistical inference
1a. Baseline (as in Fig. 1)	149.40	18	8.30	< 0.01	0.16	0.14 to 0.18	0.88	0.71	0.13			
1b. Baseline (as in Fig. 2)	141.93	19	7.47	< 0.01	0.15	0.13 to 0.17	0.89	0.74	0.12			
1c. Baseline (as in Fig. 2)	77.97	15	5.20	< 0.01	0.12	0.09 to 0.15	0.94	0.83	0.05			
1d. Baseline (saturated-model)	0.00	0	–	< 0.01	0.00	0.00 to 0.00	1.00	1.00	0.00			
1e. Baseline (constricted paths between IDs and PF/QL)	14.68	10	1.47	0.14	0.04	0.00 to 0.08	1.00	0.98	0.02	14.68	10	The more constricted model can be retained without significant loss of model fit
1 f. Baseline (model 1e plus additional constricted paths between IDs and TE)	19.57	14	1.40	0.14	0.04	0.00 to 0.07	1.00	0.98	0.02	19.57	14	The more constricted model can be retained without significant loss of model fit
2a. Follow-up (as in Fig. 1)	71.00	18	3.94	< 0.01	0.10	0.08 to 0.13	0.95	0.86	0.09			
2b. Follow-up (as in Fig. 2)	109.02	19	5.74	< 0.01	0.13	0.10 to 0.15	0.91	0.78	0.09			
2c. Follow-up (as in Fig. 2)	101.39	15	6.76	< 0.01	0.14	0.12 to 0.17	0.91	0.73	0.07			
2d. Follow-up (saturated-model)	0.00	0	–	< 0.01	0.00	0.00 to 0.00	1.00	1.00	0.00			
2e. Follow-up (constricted paths between IDs and PF/QL)	12.71	10	1.27	0.24	0.03	0.00 to 0.07	1.00	0.99	0.02	12.71	10	The more constricted model can be retained without significant loss of model fit
2 f. Follow-up (model 1e plus additional constricted paths between IDs and TE)	18.19	14	1.30	0.20	0.03	0.00 to 0.07	1.00	0.99	0.02	18.19	14	The more constricted model can be retained without significant loss of model fit

*χ*^*2*^ Chi square statistic, *df* degrees of freedom, *RMSEA* root mean square error of approximation, *CFI* comparative fit index, *TLI* Tucker-Lewis Index, *SRMR* standardized root mean square residual, *Δ χ*^*2*^ Chi square value of the MLR difference test, *Δ df* difference in degrees of freedom between the models being compared, *IDs* internal determinants, *PF* psychosocial functioning, *QL* quality of life, *TE* treatment engagement

**Fig. 2:**
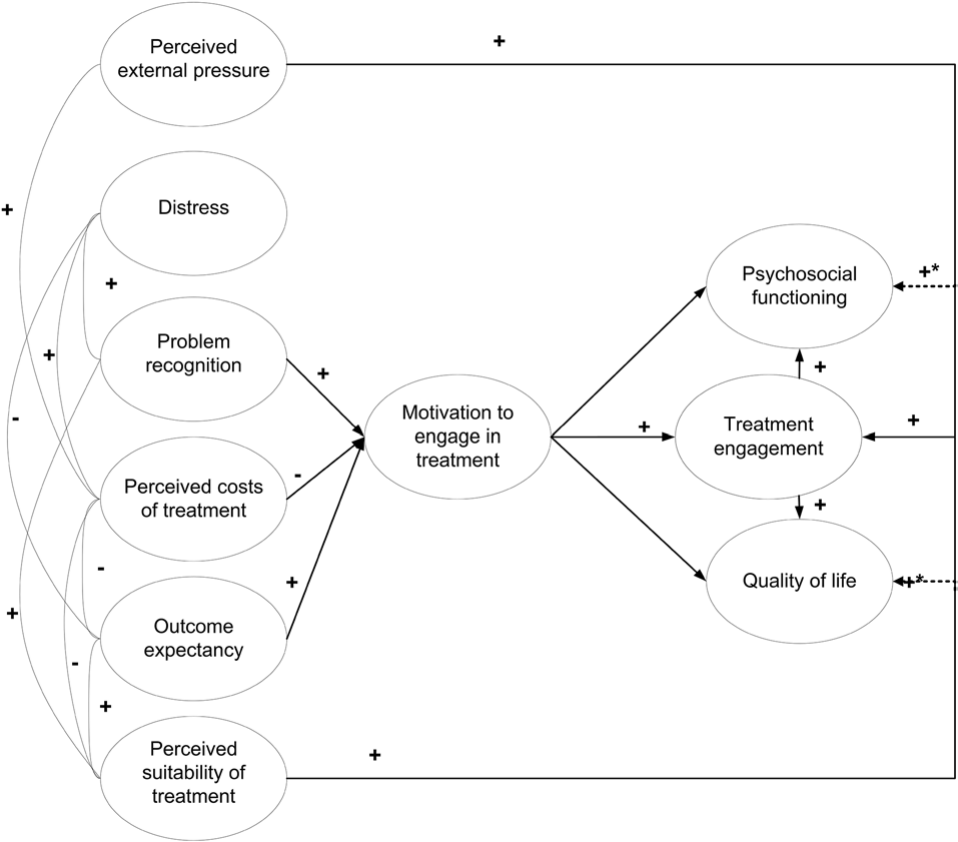
The process model based on Drieschner and Boomsma (2008b). *Note* The figure represents models 1b/2b. The dotted lines (indicated by *) represent regression estimates that were added in a second version to create models 1c/2c, in which psychosocial functioning and quality of life were also determined by suitability of treatment and external pressure directly. The figure depicts latent variables, the observed variables and accompanying measurement errors underlying the latent variables were left out to avoid a cluttered presentation

In search of a more plausible model, modification indices and, in particular, standardized residuals were inspected. These did not provide theoretically plausible nor unequivocal suggestions for improving model fit. That is, the indices pointed to several lacking direct effects between internal determinants and clinical outcomes (treatment engagement, psychosocial functioning and quality of life), some of which were opposite to theoretical expectations. Therefore, it was decided to investigate whether the structural model that was empirically obtained by Drieschner and Boomsma (2008b) would show better fit to the data than the originally hypothesized model (2004).

The empirically derived model by Drieschner and Boomsma (2008b) was tested at both time points and labelled as model 1b (baseline) and model 2b (follow-up) in Table 3. This model is shown in Fig. 2 and included indirect paths from problem recognition, outcome expectancy and costs of treatment to treatment engagement via MET (while the paths between the remaining three internal determinants and MET were constrained to 0), and direct paths from suitability of treatment, external pressure and MET to treatment engagement. As model fit was not good, modification indices and standardized residuals were inspected. These suggested that there should be direct paths from suitability of treatment and external pressure to psychosocial functioning and quality of life, as shown by the dotted lines in Fig. 2 (models 1c and 2c). The results in Table 3 show that model fit improved slightly but remained borderline for baseline assessment (models 1b and 1c), whereas it became worse for the follow-up assessment (models 2b and 2c) compared to the model depicted in Fig. 1. Thus, these models did not show acceptable fit to the data.

In search for a more plausible model, a model was tested which included paths from all predictors to all subsequent variables in the model (i.e. resulting in 0 degrees of freedom). By definition, the fit of this model (which we labelled ‘saturated-model’) was perfect for both assessment moments (see models 1d and 2d in Table 3). Subsequently, a backward elimination procedure was applied to the saturated-model to obtain a more constrained model while not statistically significantly reducing model fit. The MLR χ^2^ difference test was used to compare nested rivalling models on model fit. The backward procedure started with the constriction of paths from the internal determinants to the distal outcomes (psychosocial functioning and quality of life) as these paths were least in line with theory (Drieschner et al. 2004). Specifically, the regression paths were sequentially constrained to zero between each internal determinant and the two distal outcomes to determine which constrictions were acceptable, i.e. did not statistically significantly reduce model fit. It was found that all paths between the internal determinants and two distal outcomes could be constrained to zero except for the path between distress and both outcomes. The fit for this model for both assessment moments is presented in Table 3 (models 1e and 2e) and also shows the results of the MLR χ^2^ difference test between the saturated-model (models 1d and 2d) and the constrained models (models 1e and 2e).

**Fig. 3:**
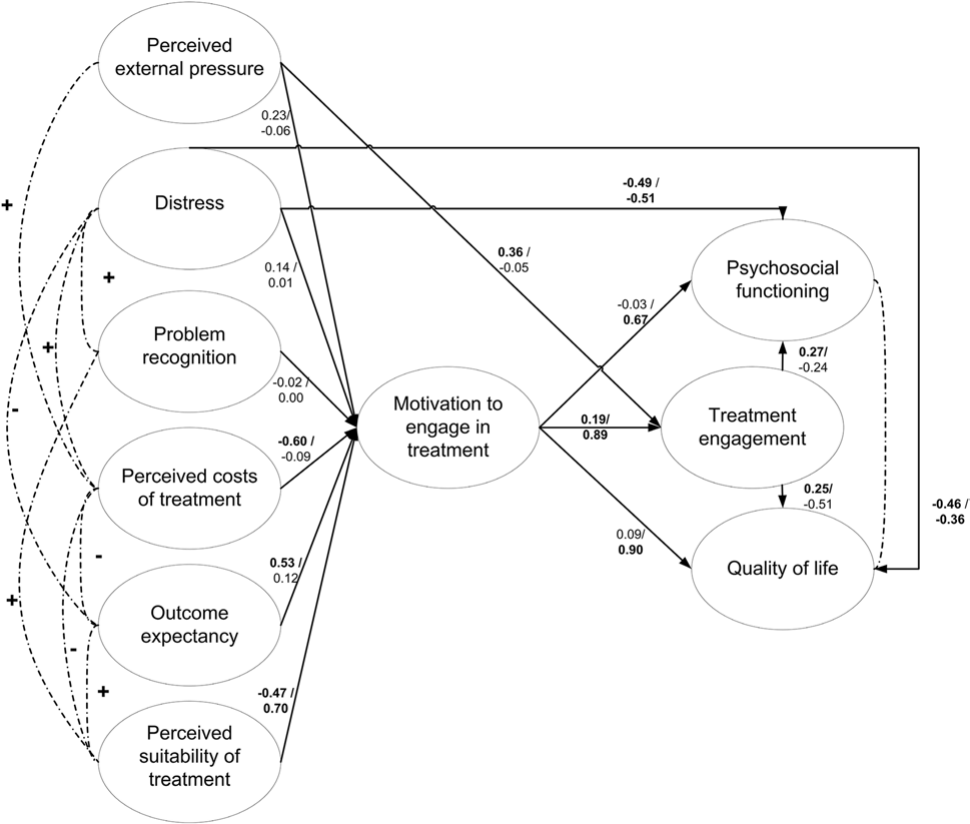
Testing the obtained process model for IM across time on clinical outcomes. *Note* The figure represents Model 3a, with all regression coefficients left unconstrained for the corresponding measurements at baseline and follow-up (i.e. indicating that these are variant across time). Numbers represent standardized regression coefficients for the corresponding path (baseline / follow-up). Thick lines represent regression paths, dotted lines represent intercorrelations of variables. Boldface indicates statistical significance at *P* < 0.05 (two-tailed). The figure depicts latent variables, the observed variables and accompanying measurement errors underlying the latent variables were left out to avoid a cluttered presentation

Subsequently, it was investigated if the path from MET to the distal outcomes could be constrained to zero, which was acceptable for the baseline model but not for the follow-up assessment. It was therefore decided to retain this path in the model unconstrained. Then, it was investigated which paths between the internal determinants to treatment engagement could be constrained to zero without significant loss of model fit. It was found that all paths from internal determinants to treatment engagement could be constrained to zero, except for the paths from distress and external pressure to treatment engagement (see Table 3 for the MLR χ^2^ difference test between the saturated-model and models 1f and 2f). This model was accepted as the final model, as further constrictions (e.g. between the IDs and MET) would hinder the testing of the ‘core’ of the original theory which consists of the mediating role of motivation between the six internal determinants and treatment engagement (Drieschner et al. 2004). Figure 3 shows the accepted final structural model including the standardized regression coefficients for all paths in the model, in which it can be seen that the strongest positive associations were found from perceived suitability of treatment, perceived costs of treatment and outcome expectancy to motivation for engaging in treatment, whereas strong negative associations were found between distress and treatment engagement, psychosocial functioning and quality of life.

#### Test of the IM process model across time and across patient groups

Subsequently, the best-fitting model (1f/2f) was tested across time and across patient groups. Testing the obtained process model across time was done by testing the invariance of the regression estimates of the latent variables across the two measurement occasions. A model was created in which both baseline and follow-up latent path models were included simultaneously (Model 3). In the first version of this model the regression weights were allowed to be free (unconstrained) for the baseline and follow-up measurements (Model 3a), which resulted in borderline fit to the data (χ^2^/df = 2.85, RMSEA = 0.08, CFI = 0.94, TLI = 0.86, SRMR = 0.08). Then, a second version of this model was created in which the regression weights for the corresponding paths at baseline and follow-up were constrained to be similar (Model 3b). Compared to Model 3a, Model 3b provided much worse fit to the data (χ^2^/df = 5.84, RMSEA = 0.13, CFI = 0.80, TLI = 0.63, SRMR = 0.17). The test for invariance across time was represented by the MLR χ^2^ difference test between Models 3a and 3b, where a non-significant χ^2^-test was considered statistical evidence for the latent path model being invariant across time. As can be seen in Table 4, the χ^2^-test reached statistical significance (Δχ^2^ = 247.47, Δdf = 15, *P* = < 0.01), implying that the IM model was not invariant across time. That is, the regression coefficients between variables in the model could not be considered similar for the baseline and follow-up assessments, as at least some of these were significantly different for the two time points. Model 3a is shown in Fig. 2, including standardized regression coefficients for the baseline and follow-up measurements.

**Table 4:** Model comparisons to test for robustness of the obtained model across time and patient groups

Model	C or U	χ^2^	df	χ^2^/ df	Δ Χ^2^	Δ df	Δ Χ^2^/Δ df	*P* value	Interpretation based on statistical inference
3a. Baseline and follow-up jointly (as 1f and 2f)	U	244.94	86	2.85	247.47	15	16.50	< 0.01	The model is variant across time
3b. Baseline and follow-up jointly (as 1f and 2f)	C	589.39	101	5.84					
4a. Baseline process model (as 1f) for psychotic versus personality disorders	U	31.11	28	1.11	17.57	15	1.17	0.29	The model is invariant across patient groups at baseline
4b. Baseline process model (as 1f) for psychotic versus personality disorders	C	48.92	43	1.13					
5a Follow-up process model (as 2f) for psychotic versus personality disorders	U	50.67	28	1.81	38.00	15	2.53	< 0.01	The model is variant across patient groups at follow-up
5b Follow-up process model (as 2f) for psychotic versus personality disorders	C	87.25	43	2.03					

*C or U* Model with either constrained (C) or unconstrained (U) regression coefficients for corresponding measurements at baseline and follow-up. The constrained (nested) model is the more constrictive model with more degrees of freedom than the comparison model. The grey and white shading indicates models that are rivalling (nested) models (similar shading indicates rivaling models)

*χ*^*2*^ Chi square statistic, *df* degrees of freedom, *Δ χ*^*2*^ Chi square value of the MLR difference test, *Δ df* difference in degrees of freedom between the models being compared

Additionally, it was tested whether the IM model was invariant across different patient groups. To this end, the IM model was tested for differences between the group of patients with a primary diagnosis and patients with a primary diagnosis of a personality disorder. First, it was tested whether this model at baseline (Model 1f) was invariant across patient groups by evaluating the χ^2^-difference test, which compared the model with all corresponding regression estimates constrained to be equal for the two patient groups (Model 4b) to the model where all regression estimates were unconstrained for the two patient groups (Model 4a). Table 4 shows the results for this comparison and it can be seen that the χ^2^-test did not reach statistical significance (Δχ^2^ = 17.57, Δdf = 15, *P* = 0.29), which provided support for the hypothesis that the IM model was invariant across these different patient groups at the baseline measurement.

The same procedure was repeated for the IM process model at follow-up. Here, it was found that the χ^2^-test for nested models did reach statistical significance (Δχ^2^ = 38.00, Δdf = 15, *P* < 0.01), which was interpreted as the IM process model being not invariant across the patient groups at follow-up. That is, although the two patient groups could be described by a similar structural model at baseline (i.e. the regression coefficients between variables in the model at baseline were not significantly different between the groups), this was not the case for the follow-up assessment. Further testing of differences between patient groups with models that included both time points simultaneously also showed that the two patient groups were not invariant. In sum, these tests of the obtained IM process model suggest that this model is not stable across time nor across patient groups.

#### Variance explained and predictive value of the IM process model

It can be seen in Table 5 that the obtained IM process model explained between 22 and 86% of treatment engagement, between 38 and 43% of psychosocial functioning and between 31 and 42% of quality of life, depending on the timing of the assessment.

**Table 5:** Variances explained by the IM process model

Model	Variance (R^2^)			
	MET	TE	PF	QL
1. Baseline	0.44	0.22	0.38	0.42
2. Follow-up	0.73	0.86	0.43	0.31

*MET* Motivation to engage in treatment, *TE* treatment engagement, *PF* Psychosocial functioning, *QL* quality of life, *N.a*. not applicable. Boldface indicates *P* < 0.05 (two-tailed)

## Discussion

### Key findings and interpretation

Regarding the first objective, the hypothesized mediational effect of motivation for engaging in treatment between internal determinants and treatment engagement was only partially supported. A mediation effect was only found for the variables perceived problem recognition, perceived suitability of treatment, perceived costs of treatment and perceived outcome expectancy, whose effects on treatment engagement were mediated by motivation for treatment (see Fig. 3). However, no such full mediation effect was found for distress and perceived external pressure. The model did not show a good model fit until additional direct paths between distress and all clinical outcomes were incorporated. Perceived external pressure was found to be of direct influence on the patient’s treatment engagement, independent of a mediational effect by motivation. Thus, the final structural model was neither in line with original hypothesized theory as shown in Fig. 1 nor was it similar to the obtained empirical model which was previously found by Drieschner and Boomsma in a forensic psychiatric research population (2008b), in which the patient’s motivation for engaging in treatment also mediated the relation between problem recognition and treatment engagement (whereas we found no effect of problem recognition on any of the outcomes) and which showed that suitability of treatment was directly related to treatment engagement (whereas we found a mediational effect) and showed no effect of perceived costs of treatment (whereas we did).

Regarding the second objective, the obtained plausible model was not stable across time nor across different patient groups. These findings indicate that this theory in its current form does not constitute a robust framework for patterns through which patients become motivated to engage in treatment. On the one hand, it is not surprising that the identified model differs between patients with psychotic disorders and personality disorders, or that this is different for forensic psychiatric outpatients compared to outpatients with severe mental illness (with or without a history of offending). On the other hand, it would have strengthened the utility and generalizability of the theory if similar patterns of associations between motivational variables would appear across time and across different patient populations. Future studies should aim to replicate the current study in other populations and aim to explain (if and) why these differences occur. In addition, since the patient’s quality of life and psychosocial functioning are of great interest to treatment outcomes, future studies may aim to explore subdomains within these outcomes and how this affects the fit of the model.

Despite these findings regarding the structure and stability of the IM, the current study does provide insight into which factors are most relevant for the patient’s motivation and treatment engagement. Both our work and that of Drieschner (2005) showed that perceived suitability of treatment and outcome expectancy were most strongly associated with motivation and treatment engagement. These determinants comprise the patient’s perception of the treatment and relationship with the clinician, and the perceived competence in being able to do what the treatment requires, and the findings underscore their importance in relation to motivation and treatment outcomes.

Further, the level of distress is generally regarded an important determinant of treatment motivation, such that more (symptomatic) suffering makes patients more motivated to engage in treatment (Angst et al. 2010). Indeed, studies have found that treatment-seeking patients with personality disorders or substance-use disorders reported higher subjective distress than those who did not seek treatment (van Beek and Verheul 2008; Velasquez et al. 2000). However, others have found a so-called ‘motivation paradox’ in patients with SMI, such that those with more symptoms and more psychosocial problems are less motivated for engaging in treatment (Mulder et al. 2014). This latter observation is consistent with the current study, where distress showed a negative association with treatment engagement and was unrelated to motivation for engaging in treatment (controlling for the other internal determinants). Drieschner and Boomsma found similar results in their studies in forensic psychiatric patients (2008b). This implies that, higher distress may withhold outpatients with SMI from in engaging with treatment, which may be related to the finding that higher distress is also associated with lower outcome expectancy and lower perceived suitability of treatment (see Table 2). For patients where distress is high and other motivational determinants are low, this may provide an argument for the paternalistic practices as performed by the assertive outreach teams, trying to engage patients who might otherwise be left untreated (Mulder et al. 2014). These patients might be engaged by first increasing the external (legal) pressure, as – again similar to the findings of Drieschner and Boomsma (2008b) - we found that perceived external (legal) pressure was directly related to treatment engagement, whereas no significant association between external pressure and motivation was found. These findings suggest that patients may engage in treatment due to external pressures, regardless of how motivated they are (by themselves). Alternatively, this finding may relate to the assessment of treatment engagement with the SES. It has been noted that the items in the SES do not constitute a complete measure of engagement that represents all efforts clients make during the course of treatment, but only reflect those that are observed by clinicians within and surrounding sessions, not those between sessions or the view of patients themselves (Holdsworth et al. 2014). The authors of the SES acknowledge that there may be an element of coercion in asking clients to engage with treatment services (Tait et al. 2002), which is also reflected in the operationalization of treatment engagement in the SES, and this may explain why external pressure as perceived by patients is related to treatment engagement and not to self-reported motivation in our study.

Regarding the differences between the structural models at the two time points, it seems remarkable that not only the strengths of the relationships between the IDs and motivation were different, but also—in some cases—the direction of these relationships. For example, the correlation between perceived suitability of treatment and motivation was positive (see Table 2), but when corrected for the influence of the other internal determinants resulted in a negative association at baseline, and again a positive association at follow-up (see Fig. 2). After ruling out the possibility of multicollinearity problems, we interpreted this finding as valid and indicating that the interrelations of the internal determinants are more complex than the current theory suggests. This should therefore be subject of subsequent investigations of the IM.

Thirdly and finally, the obtained plausible model was able to explain substantial amounts of variance in treatment engagement, psychosocial functioning and quality of life. The model explained between 22 and 86% of treatment engagement, between 38 and 43% of psychosocial functioning and between 31 and 42% of quality of life, depending on the timing of the assessment. The discrepancy between explained variances at baseline and at follow-up may be explained by the relative contributions of perceived suitability of treatment and motivation, which were more pronounced at the follow-up assessment. All in all, this suggests that the concepts contained within the IM hold potential to predict treatment outcomes, which warrants further empirical investigation into the IM.

### Strengths and limitations

Strengths of the current study include the longitudinal component which allowed for testing of the model at two time points, a relatively large sample size considering the often difficult to engage patient population, that it was a multi-center study, the correction for unreliability of measurements and testing of rivalling models.

Limiting the current study is the possibility of model misspecification, which should not be underestimated. Misspecification of the model may have occurred due to misspecification of the relations between the internal determinants or if some of the relations in the model were actually bidirectional (such as between distress and psychosocial functioning and quality of life). These alternatives were not tested as these were not in line with IM, but the idea of reciprocal relationships between some of the variables in the model is actually possible. For example, not only may motivation for engaging with treatment depend on the patient’s outcome expectancy, but in turn the patient’s outcome expectancy may depend on (previous) motivation for engaging in treatment and previous treatment engagement behaviours. Such relations are likely for ongoing, repeated behaviours (Weinstein 2007) as is the case in our study sample, where the mean age of first contact with mental health care was 26 (Jochems et al. 2015). Further, although efforts were made to compare different structural models and to identify a model which was most plausible considering both theory and data, our final model was based on a backward elimination approach which opens the possibility of a ranking and selection problem. That is, the constriction of certain paths in the model to zero (i.e. “dropping them”), was based on this study sample which might not be generalizable to other samples let alone to the entire population of outpatients with SMI. Future studies should try to replicate our findings in other samples and with more elaborate measures of treatment engagement, as engagement may differ across client groups and the differentiation between treatment engagement, treatment compliance and medication compliance is relevant for a comprehensive understanding of the relationships between internal determinants, motivation and treatment engagement. Furthermore, future studies may also want to include means into model testing, to investigate whether these are different between different patient groups and over time.

It is considered a strength that our sample largely represents a broad population of outpatients with diagnoses of psychotic and personality disorders with a variety of co-morbid psychiatric disorders, which strengthens the generalizability of the study. However, patients with relatively high levels of motivation for treatment, treatment engagement and psychosocial functioning may still have been more likely to participate in and complete the study compared to patients with low motivation, low engagement and poor functioning. Therefore, the results may not be generalizable to the entire population of outpatients with SMI, in particular those patients who are not in contact with services. Future studies should further investigate the generalizability of the TMS-f and the TMS-p to other patient populations and nationalities, as the scales and the conceptual framework of the IM may prove useful in the understanding and communication about motivation for engaging with treatment services in other mental health contexts as well.

## Conclusion and implications

The current study showed that the relations between internal determinants, motivation for engaging in treatment, treatment engagement and clinical outcomes were not consistent with the original theory, nor were they consistent across time and different patient diagnostic groups. Future studies should aim to test the IM in other clinical populations, to further specify the relations between constructs in the model and to re-specify (or reject) the initially hypothesized principles. Depending on the context of these future studies, researchers may choose to use the original TMS-f or the TMS-p. The IM might be improved by re-specifying the interrelations of the internal determinants and/or by including intermediary factors such as action planning between the level of MET and the actual treatment engagement (Jochems et al. 2011; Sutton 2008). Including such intermediary factors might create opportunities to beneficially influence the pathway to treatment engagement. The constructs in the model did show explanatory value, which demonstrates the future potential of IM (constructs) as a basis for interventions in the mental health care for outpatients with SMI. In further testing of the theory, it will become more accurate and thus more useful for application in clinical practice. Clinical implications of our findings include that perceived suitability of treatment, perceived costs of treatment and outcome expectancy currently seem the most interesting targets for interventions aimed at improving motivation and treatment engagement.

## Electronic supplementary material

Below is the link to the electronic supplementary material.

Supplementary material 1 (DOCX 57 KB)
