# Supplementary material for: Testing the Integral Model of treatment motivation in outpatients with severe mental illness
Source: Motiv Emot. 2018 Jun 15;42(6):816–30. doi: 10.1007/s11031-018-9708-0 (PMC6208637; doi:10.1007/s11031-018-9708-0)
Supplement: Supplementary file 1 — Supplementary material 1 (DOCX 57 KB) [file 11031_2018_9708_MOESM1_ESM.docx]

**SUPPLEMENTARY MATERIAL FOR:**

**Testing the Integral Model of Treatment Motivation in outpatients with severe mental illness**

***Changes to the perceived legal pressure scale such that it represents perceived external pressure***

In the original TMS-f [[1](#_ENREF_1)], perceived legal pressure is the patient’s perception of the external pressure through the legal system. As the current study aimed to explore whether the IM is also applicable outside a forensic psychiatric setting, the current study decided to adapt the construct of perceived legal pressure into a more broad perceived external pressure. This adjustment can be justified by considering that only a subgroup of outpatients with SMI will be referred to or pressured into psychiatric treatment via the legal system, while (most) others will likely experience other pressures that drive their motivation for engaging with treatment (i.e. family, friends, partner, assertive outreaching clinicians). For clarity, we will refer to the revised scale as the TMS-p instead of the TMS-f, to indicate that the revised scale may be applied in a general psychiatric (hence the “p”) population.

Table S1 shows the 9 items of the original Dutch TMS-f and how these were adapted to represent 9 Dutch items for perceived external pressure in the TMS-p. The last two columns of Table S1 show their translations in English, which were made for the purpose of this supplement only, such that non-Dutch readers can also see the precise formulation and understand their content.

**Table S1. Items of the original perceived legal pressure scale compared to the items on the perceived external pressure scale**

| Item number | Original TMS-f  (items in Dutch) | TMS-p  (items in Dutch) | Original TMS-f  (loosely translated to English for the purpose of this supplement only) | TMS-p  (loosely translated to English for the purpose of this supplement only) |
| --- | --- | --- | --- | --- |
| 5 | Als therapeuten vinden dat ik mij onvoldoende inzet, zou dat vervelende justitiële gevolgen voor mij kunnen hebben | Als therapeuten vinden dat ik mij onvoldoende inzet, zou dat vervelende gevolgen voor mij kunnen hebben. | If therapists feel that I do not show enough effort for the treatment, this could have negative legal consequences for me | If therapists feel that I do not show enough effort for the treatment, this could have negative consequences for me |
| 13 | Wanneer ik door de kliniek weggestuurd zou worden, zou ik met zekerheid problemen krijgen met justitie | Wanneer ik door de kliniek weggestuurd zou worden, zou ik met zekerheid problemen krijgen. | If the clinic would send me away, this would certainly result into problems with the legal authorities for me. | If the clinic would send me away, this would certainly result into problems for me. |
| 25 | De justitiële stok achter de deur stelt bij mij weinig voor | De stok achter de deur van anderen stelt bij mij weinig voor | The big stick of the justice department does not impress me much | The big stick of other people does not impress me much |
| 34 | Het is best wel mogelijk dat justitie een oogje zou dichtknijpen, als ik de behandeling zou afbreken | Het is best wel mogelijk dat anderen een oogje zouden dichtknijpen, als ik de behandeling zou afbreken. | It is possible that the justice department would turn a blind eye, if I were to terminate the treatment | It is possible that other people would turn a blind eye, if I were to terminate the treatment |
| 43 | De druk van justitie voel ik sterk | De druk van anderen voel ik sterk. | I feel a strong pressure from the justice department | I feel a strong pressure from others |

**Table S1 (Continued).**

| Item number | Original TMS-f  (items in Dutch) | TMS-p  (items in Dutch) | Original TMS-f  (loosely translated to English for the purpose of this supplement only) | TMS-p  (loosely translated to English for the purpose of this supplement only) |
| --- | --- | --- | --- | --- |
| 57 | De justitiële gevolgen wanneer ik de behandeling nu zou afbreken zouden wel meevallen | Wanneer ik de behandeling nu zou afbreken, zouden de gevolgen wel meevallen. | If I were to drop out of treatment right now, the legal consequences would not be so bad | If I were to drop out of treatment right now, the consequences would not be so bad |
| 66 | Als de kliniek mij voortijdig zou wegsturen, zou justitie mijn straf zeker ten uitvoer leggen | Als de kliniek mij voortijdig zou wegsturen, zou ik zeker problemen met anderen krijgen. | If the clinic were to dismiss me prematurely, the legal department would certainly follow through with my punishment | If the clinic were to dismiss me prematurely, I would certainly get into problems with others |
| 76 | Als ik niet in behandeling was gegaan, dan had dat justitiële gevolgen gehad waar ik nogal tegenop zie | Als ik niet in behandeling was gegaan, dan had dat gevolgen gehad waar ik nogal tegenop zie. | If I had not entered into treatment, it would have had legal consequences that I find dreadful | If I had not entered into treatment, it would have had consequences that I find dreadful |
| 85 | Door mijn justitiële situatie heb ik geen echte keus, ik moet de behandeling wel afmaken | Door mijn situatie heb ik geen echte keus, ik moet de behandeling wel afmaken | Because of my legal situation, I don’t really have a choice. I have to finish the treatment. | Because of my situation, I don’t really have a choice. I have to finish the treatment. |

Table S2 shows the item properties of the 9 items represented in the perceived external pressure scale. It was decided to retain all items for subsequent use in order to maximize the comparability with the original TMS-f perceived legal pressure scale.

**Table S2. Item properties of the perceived external pressure scale and factor loadings**

| Item number | Mean | Variance | Skewness  (standard error) | Kurtosis  (standard error) | Observed range | Factor Loading |
| --- | --- | --- | --- | --- | --- | --- |
| 5 | 3.18 | 2.08 | -0.17 (0.14) | -1.29 (0.29) | 1 to 5 | 0.28 |
| 13 | 2.63 | 2.28 | 0.32 (0.14) | -1.35 (0.29) | 1 to 5 | 0.58 |
| 25 | 3.53 | 1.74 | -0.50 (0.14) | -0.80 (0.29) | 1 to 5 | 0.18 |
| 34 | 3.60 | 1.69 | -0.46 (0.14) | -0.86 (0.29) | 1 to 5 | 0.21 |
| 43 | 2.74 | 2.07 | 0.28 (0.14) | -1.24 (0.29) | 1 to 5 | 0.16 |
| 57 | 3.64 | 1.74 | -0.55 (0.14) | -0.86 (0.29) | 1 to 5 | 0.56 |
| 66 | 3.28 | 2.12 | -0.33 (0.14) | -1.21 (0.29) | 1 to 5 | 0.47 |
| 76 | 1.94 | 1.35 | 1.14 (0.14) | 0.48 (0.29) | 1 to 5 | 0.54 |
| 85 | 2.53 | 2.15 | 0.45 (0.14) | -1.16 (0.29) | 1 to 5 | 0.39 |

Results for the single factor CFAs for each TMS-p scale are shown in Table S3. For all CFAs, the MLR estimation method was used. Using the criteria as outlined in the main manuscript (see Statistical Analyses), it appears that the scales showed borderline to acceptable model fit. The best fit was found for the EP-subscale, although this scale showed the lowest congeneric estimate of reliability (0.61).

**Table S3. Results of single factor CFAs for each TMS-p scale**

| Scale  (number of items) | χ^2^ | df | χ^2^/df | p-value | CFI | TLI | RMSEA | SRMR | Reliability estimate  (congeneric) |
| --- | --- | --- | --- | --- | --- | --- | --- | --- | --- |
| PR (9) | 77.77 | 27 | 2.88 | <0.001 | 0.89 | 0.85 | 0.08 | 0.06 | 0.80 |
| DS (9) | 77.00 | 27 | 2.85 | <0.001 | 0.94 | 0.92 | 0.08 | 0.04 | 0.90 |
| EP (9) | 30.86 | 27 | 1.14 | 0.2768 | 0.97 | 0.96 | 0.02 | 0.04 | 0.61 |
| CT (9) | 51.06 | 27 | 1.89 | 0.0034 | 0.93 | 0.90 | 0.06 | 0.05 | 0.78 |
| ST (9) | 57.01 | 27 | 2.11 | <0.001 | 0.94 | 0.92 | 0.06 | 0.04 | 0.86 |
| OE (9) | 79.96 | 27 | 2.96 | <0.001 | 0.91 | 0.88 | 0.08 | 0.05 | 0.86 |
| MET (16) | 216.05 | 104 | 2.08 | <0.001 | 0.83 | 0.81 | 0.06 | 0.06 | 0.82 |

In Table S4, the standardized factor loadings are shown that resulted from testing single-factor models on each of the subscales of the TMS-p, using confirmatory factor analyses (CFAs) with the robust maximum likelihood (MLR) estimation method.

**Table S4. Standardized factor loadings and residuals (standard errors) of items for each TMS-p subscale**

| Sub-scale | Item | Standar-dized factor loading | Residuals (standard errors) |
| --- | --- | --- | --- |
| PR | 2 | 0,46 | 0,78 |
|  | 19 | 0,60 | 0,63 |
|  | 78 | 0,63 | 0,60 |
|  | 11 | 0,38 | 0,85 |
|  | 26 | 0,76 | 0,41 |
|  | 36 | 0,45 | 0,79 |
|  | 49 | 0,61 | 0,62 |
|  | 58 | 0,53 | 0,71 |
|  | 70 | 0,57 | 0,67 |
| DS | 44 | 0,46 | 0,78 |
|  | 71 | 0,59 | 0,65 |
|  | 9 | 0,70 | 0,51 |
|  | 18 | 0,76 | 0,42 |
|  | 30 | 0,68 | 0,53 |
|  | 40 | 0,5 | 0,44 |
|  | 54 | 0,78 | 0,38 |
|  | 63 | 0,72 | 0,48 |
|  | 83 | 0,84 | 0,29 |
| EP | 25 | 0,18 | 0,96 |
|  | 34 | 0,21 | 0,95 |
|  | 57 | 0,55 | 0,69 |
|  | 5 | 0,28 | 0,92 |
|  | 13 | 0,59 | 0,65 |
|  | 43 | 0,16 | 0,97 |
|  | 66 | 0,47 | 0,78 |
|  | 76 | 0,54 | 0,71 |
|  | 85 | 0,39 | 0,84 |
| CT | 1 | 0,57 | 0,67 |
|  | 14 | 0,26 | 0,93 |
|  | 51 | 0,52 | 0,73 |
|  | 24 | 0,48 | 0,76 |
|  | 41 | 0,60 | 0,63 |
|  | 61 | 0,60 | 0,62 |
|  | 67 | 0,60 | 0,62 |
|  | 80 | 0,57 | 0,66 |
|  | 27 | 0,47 | 0,77 |
| Sub-scale | **Item** | **Standardized factor loading** | **Residuals (standard errors)** |
| ST | 15 | 0,66 | 0,55 |
|  | 52 | 0,63 | 0,59 |
|  | 69 | 0,59 | 0,65 |
|  | 3 | 0,75 | 0,43 |
|  | 21 | 0,73 | 0,45 |
|  | 33 | 0,68 | 0,53 |
|  | 47 | 0,62 | 0,61 |
|  | 62 | 0,51 | 0,73 |
|  | 79 | 0,55 | 0,69 |
| OE | 6 | 0,54 | 0,70 |
|  | 12 | 0,75 | 0,42 |
|  | 35 | 0,73 | 0,45 |
|  | 39 | 0,75 | 0,42 |
|  | 53 | 0,61 | 0,61 |
|  | 64 | 0,53 | 0,71 |
|  | 77 | 0,67 | 0,54 |
|  | 84 | 0,66 | 0,55 |
|  | 46 | 0,39 | 0,84 |
| MET | 17 | 0,50 | 0,74 |
|  | 28 | 0,44 | 0,80 |
|  | 37 | 0,54 | 0,70 |
|  | 42 | 0,54 | 0,70 |
|  | 48 | 0,52 | 0,73 |
|  | 55 | 0,53 | 0,71 |
|  | 68 | 0,51 | 0,73 |
|  | 82 | 0,44 | 0,79 |
|  | 10 | 0,40 | 0,83 |
|  | 23 | 0,42 | 0,82 |
|  | 31 | 0,55 | 0,69 |
|  | 73 | 0,48 | 0,77 |
|  | 7 | 0,44 | 0,80 |
|  | 20 | 0,27 | 0,92 |
|  | 60 | 0,54 | 0,70 |
|  | 74 | 0,41 | 0,82 |

Note: PR = problem recognition, DS = distress, EP = external pressure, CT = perceived costs of treatment, ST = perceived suitability of treatment, OE = outcome expectancy, MET = motivation to engage in treatment.

In the next step, a CFA was performed including all six internal determinants as predictors for motivation to engage in treatment and including correlations between the internal determinants as specified by Drieschner and Boomsma [[2](#_ENREF_2)]. In line with Drieschner and Boomsma [[2](#_ENREF_2)], *“we followed the recommendation of Beauducel and Wittmann [*[*3*](#_ENREF_3)*] to give priority to the combination of the SRMR and RMSEA. This recommendation was based on the finding that in models with low or moderate factor loadings, incremental fit indices such as the TLI and the CFI penalize unspecified small secondary factor loadings (i.e., loadings on unintended factors) to a degree that such models “would only have a chance to be accepted when incremental fit indexes and the GFI are not used for model evaluation” (p. 70). In multidimensional questionnaires, secondary factor loadings are hardly avoidable, if only because of formal or linguistic features shared by items of different scales”*(p. 10).

The CFA including all six IDs as predictors for MET, showed an acceptable combination of RMSEA <0.06 and SRMR <.10, while CFI and TLI were low (χ2/df=1.71, RMSEA=0.05, CFI=0.74, TLI=0.73, SRMR=0.09). These fit indices of RMSEA and SRMR were comparable to those found by Drieschner and Boomsma[[2](#_ENREF_2)] , while the CFI and TLI were found to be somewhat lower in the current study. In line with the choices made by Drieschner and Boomsma [[2](#_ENREF_2)], it was decided that the current results of the CFAs justified using this model for the TMS-p for subsequent analyses.

In Table S5, the Pearson correlations between the observed sum scores on the subscales of the TMS-p as found in the current study are shown below the diagonal (i.e. lower triangle). For comparison, the correlations found in the study by Drieschner and Boomsma [[2](#_ENREF_2)] using the TMS-f are shown above the diagonal (i.e. upper triangle). Correlations in our study that appeared to stand out (regarding strength and direction) from those found by Drieschner and Boomsma were those between Problem Recognition and External Pressure (r=0.54 versus r=-0.12) and between Distress and External Pressure (r=0.28 versus r=-0.15), respectively. These substantial differences may be explained by changes in the External Pressure -scale, such that external pressure is more closely affiliated with problem recognition than legal pressure. For example, a patient who is convinced that leaving the treatment prematurely will lead to certain negative consequences (e.g. problems with partners, family, friends or in general) may be more likely to also recognize that there are problems for which psychiatric treatment is indicated, maybe more so than a patient who is merely pressured into treatment by the legal system. A similar line of reasoning may apply to the higher correlation that was found in the current study between Distress and External Pressure, compared to Drieschner and Boomsma, as it can be argued that patients who expect that they will experience negative consequences in multiple life-domains (e.g. problems with relatives and friends) if they were to leave the treatment prematurely, may be more likely to also experience higher levels of distress, compared to a patient who expects negative consequences in the legal domain only.

Similar to the findings of Drieschner and Boomsma, motivation was strongly correlated with the subscales costs of treatment, sutability of treatment and outcome expectancy. It should be noted that the apparently different directions of associations between the costs of treatment-scale in our study compared to Drieschner and Boomsma, have to be attributed to differences in coding (i.e. we coded this subscale such that a higher score represents higher perceived costs of treatment). As such, in the interpretation of the associations with the costs of treatment -scale, these differences in direction between the studies should be ignored.

**Table S5. Pearson correlations between observed sum scores on subscales of the TMS-p/TMS-f**

|  | PR | DS | CT | ST | OE | LP | MET |
| --- | --- | --- | --- | --- | --- | --- | --- |
| PR |  | 0.60 | 0.08 | 0.32 | -0.04 | -0.12 | 0.18 |
| DS | **0.54** |  | -0.19 | -0.04 | -0.45 | -0.15 | -0.16 |
| CT | 0.02 | **0.35** |  | 0.44 | 0.43 | -0.13 | 0.36 |
| ST | **0.12** | **-0.34** | **-0.59** |  | 0.65 | 0.06 | 0.50 |
| OE | -0.11 | **-0.55** | **-0.61** | **0.68** |  | 0.12 | 0.61 |
| EP | **0.54** | **0.28** | 0.01 | **0.24** | 0.08 |  | 0.06 |
| MET | 0.10 | **-0.18** | **-0.50** | **0.38** | **0.51** | 0.07 |  |

Below diagonal: correlations found in the current study. Above diagonal: correlations found in the study by Drieschner and Boomsma [[2](#_ENREF_2)]. PR = problem recognition; DS = distress; CT = perceived Costs of the Treatment; ST = perceived Suitability of the Treatment; OE = outcome expectancy; LP = perceived Legal Pressure; EP = perceived external pressure, MET = Motivation to Engage in the Treatment. Boldface indicates that correlation is significant at the 0.05 level (two-tailed).

Descriptive statistics of the subscales of the TMS-p are shown in Table S6. Again for comparison, both the distribution of the sum scores on the TMS-p scales of our sample and the total sample used by Drieschner and Boomsma using the TMS-f are shown.

**Table S6. Descriptive statistics of the observed sum scores on the subscales of the TMS-p**

| **Scale** | **Statistic** | **Our sample**  **(TMS-p)**  **N = 294** | **Drieschner and Boomsma**  **(TMS-f)**  **N = 376** |
| --- | --- | --- | --- |
| **PR** | Mean  S.D.  Skewness (standard error)  Kurtosis (standard error) | 30.25  7.75  -0.23 (0.14)  -0.51 (0.28) | 32.50  7.76  -0.68  0.07 |
| **DS** | Mean  S.D.  Skewness (standard error)  Kurtosis (standard error) | 25.70  9.61  0.09 (0.14)  -1.01 (0.28) | 27.11  8.83  -0.06  -0.84 |
| **CT** | Mean  S.D.  Skewness (standard error)  Kurtosis (standard error) | 34.06  6.88  -0.52 (0.14)  -0.14 (0.28) | 32.85  6.91  -0.38  -0.33 |
| **ST** | Mean  S.D.  Skewness (standard error)  Kurtosis (standard error) | 34.96  7.17  -0.36 (0.14)  -0.49 (0.28) | 35.67  6.72  -0.92  1.01 |
| **EP / LP** | Mean  S.D.  Skewness (standard error)  Kurtosis (standard error) | 30.41  5.96  -0.06 (0.14)  -0.18 (0.28) | 24.00  9.36  0.15  -1.00 |
| **MET** | Mean  S.D.  Skewness (standard error)  Kurtosis (standard error) | 47.23  11.74  0.08 (0.14)  0.08 (0.28) | 51.65  12.39  -0.19  -0.26 |

**References**

1. Drieschner, K.H. and A. Boomsma, *The Treatment Motivation Scales for forensic outpatient treatment (TMS-F): construction and psychometric evaluation.* Assessment, 2008. **15**(2): p. 224-241.

2. Drieschner, K.H. and A. Boomsma, *Validation of the Treatment Motivation Scales for Forensic outpatient treatment (TMS-F).* Assessment, 2008. **15**(2): p. 242-255.

3. Beauducel, A. and W.W. Wittmann, *Simulation study on fit indices in confirmatory factor analysis based on data with slightly distorted simple structure.* Structural Equation Modeling, 2005. **12**: p. 41-75.
